# Correction: Hybrid cellulose nanocrystal/alginate/gelatin scaffold with improved mechanical properties and guided wound healing

**DOI:** 10.1039/d2ra90124b

**Published:** 2022-12-12

**Authors:** Yue Shan, Chaoyue Li, Yongzhi Wu, Qiwen Li, Jinfeng Liao

**Affiliations:** State Key Laboratory of Oral Diseases, National Clinical Research Centre for Oral Diseases, West China Hospital of Stomatology, Sichuan University Chengdu 610041 China liaojinfeng.762@163.com

## Abstract

Correction for ‘Hybrid cellulose nanocrystal/alginate/gelatin scaffold with improved mechanical properties and guided wound healing’ by Yue Shan *et al.*, *RSC Adv.*, 2019, **9**, 22966–22979, https://doi.org/10.1039/C9RA04026A.

The authors regret that incorrect versions of Fig. 7 and 8 were included in the original article. The correct versions of Fig. 7 and 8 are presented below.

**Fig. 7:**
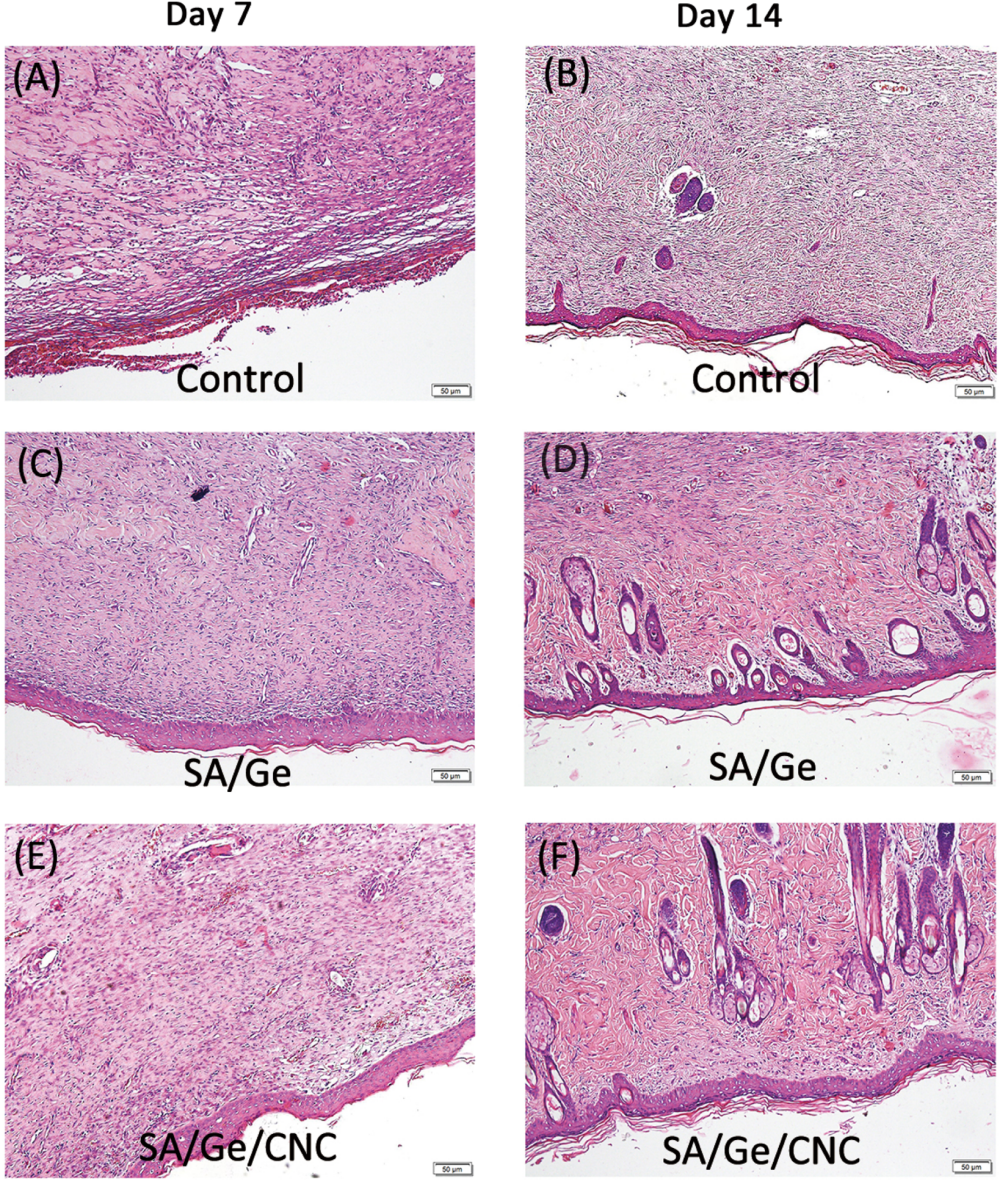
H&E staining images in control, SA/Ge, and SA/Ge/CNC groups at 7 days and 14 days after surgery. The bar corresponds to 50 μm.

**Fig. 8:**
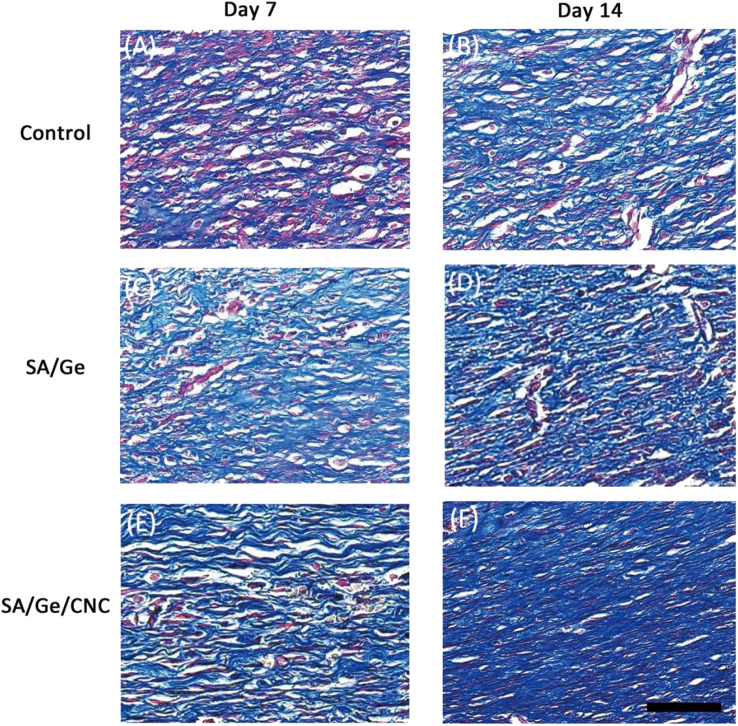
Masson’s trichrome staining images in control, SA/Ge, and SA/Ge/CNC groups at 7 and 14 days after surgery. The bar corresponds to 50 μm.

An independent expert has viewed the corrected images/data and has concluded that they are consistent with the discussions and conclusions presented.

The Royal Society of Chemistry apologises for these errors and any consequent inconvenience to authors and readers.

## Supplementary Material

